# Metabolic Response of *Faecalibacterium prausnitzii* to Cell-Free Supernatants from Lactic Acid Bacteria

**DOI:** 10.3390/microorganisms8101528

**Published:** 2020-10-05

**Authors:** Mathilde Lebas, Peggy Garault, Daniel Carrillo, Francisco M. Codoñer, Muriel Derrien

**Affiliations:** 1 Danone Nutricia Research, RD 128 Avenue de la Vauve, 91767 Palaiseau Cédex, France; lebasmat@gmail.com (M.L.); peggy.garault@danone.com (P.G.); 2 Archer Daniels Midland Co-Biopolis ADM Nutrition, C/Catedratico Agustin Escardino num 9 Edif 2, Paterna, 46980 Valencia, Spain; dacaba.92@gmail.com; 3 Present Address: Danone Nutricia Research, 30th Biopolis Street Matrix Building, Singapore 138671, Singapore

**Keywords:** *Faecalibacterium prausnitzii*, lactic acid bacteria, lysis, in vitro

## Abstract

Interest in preventive or therapeutic strategies targeting gut microbiota is increasing. Such strategies may involve the direct replenishment of the gut microbiota with single strains or strain mixtures, or the manipulation of strain abundance through dietary intervention, including lactic acid bacteria. A few candidate species associated with health benefits have been identified, including *Faecalibacterium prausnitzii*. Given its growth requirements, modulation of this bacterium has not been extensively studied. In this investigation, we explored the capacity of cell-free supernatants of different *Lactobacillus*, *Streptococcus*, *Lactococcus,* and *Bifidobacterium* strains to stimulate the growth of *F. prausnitzii* A2-165. Modulation by four strains with the greatest capacity to stimulate growth or delay lysis, *Lactococcus lactis* subsp. lactis CNCM I-1631, *Lactococcus lactis* subsp. *cremoris* CNCM I-3558, *Lactobacillus paracasei* CNCM I-3689, and *Streptococcus thermophilus* CNCM I-3862, was further characterized by transcriptomics. The response of *F. prausnitzii* to cell-free supernatants from these four strains revealed several shared characteristics, in particular, upregulation of carbohydrate metabolism and cell wall-related genes and downregulation of replication and mobilome genes. Overall, this study suggests differential responses of *F. prausnitzii* to metabolites produced by different strains, providing protection against cell death, with an increase in peptidoglycan levels for cell wall formation, and reduced cell mobilome activity.

## 1. Introduction

*Faecalibacterium prausnitzi* (*F. prausnitzii*) is a Gram-negative bacterium that is prevalent and abundant in healthy subjects [1]. It produces butyrate, as a major fermentation end-product, which acts as an energy source for colonocytes. Its presence is generally considered beneficial to intestinal health [2,3]. Interest in *F. prausnitzii* has increased over the last decade, mostly in response to the pioneering study by Sokol et al. reporting a depletion of *F. prausnitzii* in Crohn’s disease patients [4]. Since this report, many studies have confirmed that changes in the abundance of *F. prausnitzii* occur in various other diseases (reviewed by [5]). Depletion of *F. prausnitzii* in inflammatory conditions may reflect the sensitivity of this bacterium to the high luminal oxygen concentrations resulting from inflammation. *F. prausnitzii* has been reported to have anti-inflammatory effects in vivo, in models of acute [4] or chronic [6] chemically induced active inflammation and in conditions of low-grade inflammation [7], mediated in part by the secreted metabolites blocking the activation of nuclear factor (NF)-κB [8]. A 15 kDa candidate protein (microbial anti-inflammatory molecule, MAM) was identified and shown to alleviate chemically-induced colitis in mice [9]. These studies support the notion that *F. prausnitzii* contributes to immune homeostasis in the intestine via its anti-inflammatory activities [10] and suggest that it could be considered a biomarker of healthy ecosystems [11]. A recent study reported co-abundance between *F. prausnitzii* and multiple species in IBD including negative relationship with *Haemophilus parainfluenzae* [12].

*F. prausnitzii* strains are diverse, and two to three phylogroups have been defined [13,14] on the basis of 16S rRNA sequence analysis. These phylogroups were shown to be independent of substrate use, pH tolerance, or bile sensitivity [15]. A recent analysis of 31 genomes identified two “genomogroups” differing in terms of their carbohydrate and amino-acid metabolism and their defense mechanisms [16].

The global nutritional needs of *F. prausnitzii* have been investigated through a manually curated metabolic reconstruction that identified several amino acids and vitamins as growth factors [17]. In vitro experiments have shown that *F. prausnitzii* can metabolize fibers, directly or indirectly, through metabolic cross-feeding. Acetate consumption is the major driver of butyrate production by *F. prausnitzii* [18]. The consumption of acetate released by *Bifidobacterium adolescentis* has been shown to stimulate *F. prausnitzii* when these two species are grown together on fructo-oligosaccharides [19]. *F. prausnitzii* can metabolize substrates from both host and dietary sources, with some variability between strains [13]. One of these substrates, apple pectin, has been reproducibly reported to favor an increase in *F. prausnitzii* abundance in monoculture [20], complex communities [21,22] and humans [23].

Lactic acid bacteria/bifidobacteria consumption have been shown to modulate gut microbiota [24]). In vitro models have allowed to identify cross-feeding between lactic acid bacteria/bifidobacteria and gut microbiota through short-chain fatty acids (SCFAs) [25,26], cell wall-derived polysaccharides [27,28], and vitamins [29]. Many studies have reported then capacity of lactic acid bacteria to inhibit pathogen growth [30,31] through the production antimicrobial compounds such as bacteriocins [32]. However, the ability of lactic acid bacteria and bifidobacteria to modulate the growth and metabolism of strains of potential interest for human health has been little explored. In this study, we investigated the response of *F. prausnitzii* A2-165 (DSM 17677) to cell-free supernatants from selected lactic acid bacteria and bifidobacteria in vitro.

## 2. Materials and Methods

### 2.1. Bacterial Strains and Media

*Faecalibacterium prausnitzii* A2-165 was purchased from DSMZ, Braunschweig, Germany (DSM 17677). It was routinely grown in brain–heart infusion (BHI) medium (Becton Dickinson, Le Pont-de-Claix, France) supplemented with 0.5% yeast extract (Becton Dickinson, Le Pont-de-Claix, France) and 5 mg/L hemin chloride (Calbiochem, VWR International, Libourne, France), with cellobiose (1 g/L; Sigma-Aldrich Co. LLC, St. Louis, MO, USA), maltose (1 g/L; Sigma-Aldrich Co. LLC, St. Louis, MO, USA), and cysteine (0.5 g/L; Sigma-Aldrich Co. LLC, St. Louis, MO, USA), under an atmosphere consisting of 80% N_2_–10% CO_2_–10% H_2_. Thirteen lactic acid bacteria/bifidobacteria strains were tested for their impact on growth of *F. prausnitzii* (Table S1). They were maintained in medium M17 lactose (*Streptococcus* and *Lactococcus*) or MRS plus 1 g/L l-cysteine (*Bifidobacterium*) or MRS (*Lactobacillus*), AES Laboratoire, Combourg, France.

To test the effect of supernatants on the growth of *F. prausnitzii*, all strains were cultured in YCFA medium optimized for the growth of all bacterial strains. This medium consisted of glucose (2 or 20 g/L, Sigma-Aldrich Co. LLC, St. Louis, MO, USA), tryptone (10 g/L, Becton Dickinson, Le Pont-de-Claix, France), yeast extract (5 g/L, Becton Dickinson, Le Pont-de-Claix, France), sodium acetate (5 g/L, Sigma-Aldrich Co. LLC, St. Louis, MO, USA), monohydrate lactose (5 g/L, Sigma-Aldrich Co. LLC, St. Louis, MO, USA), sodium bicarbonate (4 g/L, Sigma-Aldrich Co. LLC, St. Louis, MO, USA), cellobiose (2 g/L, Sigma-Aldrich Co. LLC, St. Louis, MO, USA), sodium chloride (0.9 g/L, Sigma-Aldrich Co. LLC, St. Louis, MO, USA), ammonium sulfate (0.9 g/L, Sigma-Aldrich Co. LLC, St. Louis, MO, USA), cysteine (0.5 g/L, Sigma-Aldrich Co. LLC, St. Louis, MO, USA), dibasic potassium phosphate (0.45 g/L, Sigma-Aldrich Co. LLC, St. Louis, MO, USA), magnesium sulfate (0.09 g/L, Sigma-Aldrich Co. LLC, St. Louis, MO, USA), calcium chloride (0.09 g/L, Sigma-Aldrich Co. LLC, St. Louis, MO, USA), hemin chloride (0.01 g/L Sigma-Aldrich Co. LLC, St. Louis, MO, USA), and resazurin sodium salt (0.001 g/L, Alfa Aesar, Kandel, Germany). Vitamins and volatile fatty-acid (except acetate) pools were removed to limit *F. prausnitzii* growth. We also increased the buffering of the medium, to prevent excessive pH variation that might not be tolerated by the commensal bacteria. We supplemented the medium with lactose (0.5%) to support the growth of the tested lactic acid bacteria and bifidobacterial strains. Final pH was 5.7 to mimic proximal gastrointestinal conditions. This modified medium is referred to as YCFAm.

### 2.2. Culture of F. prausnitzii A2-165 with Supernatants of Lactic Acid Bacteria and Bidifobacteria

Supernatants were produced as follows: strains were sub-cultured twice in YCFAm before being inoculated in 50 mL of YCFAm at 37 °C for 24 h. Cultures were pelleted by centrifugation at 7500× *g* for 10 min, and the supernatants were passed through filters with 0.2 µm pores and directly frozen at −20 °C. Before use, supernatants were reduced by incubation in an anaerobic chamber for three hours. Reduced control medium or supernatant from each of 13 bacterial strains was added to YCFAm (8% *v*/*v*) for inoculation of a 48-h culture of *F. praunistzii,* at a concentration of 1%. OD_600_ and pH were measured at regular intervals during the growth period of 72 h.

### 2.3. Analysis of Short Chain Fatty Acids

We collected 2 mL of fresh culture, which we then centrifuged at 10,000× *g* for 15 min. The supernatants were filtered (filter with 0.2 µm pores) and stored at −20°C until analysis. Samples for short-chain fatty acid (SCFA) analyses were collected after 16, 22, and 40 h. SCFAs were extracted from the samples in diethyl ether, after the addition of 2-methyl hexanoic acid as an internal standard. Extracts were analyzed with a GC-2014 gas chromatograph (Shimadzu, ’s-Hertogenbosch, The Netherlands), equipped with a capillary fatty acid-free EC-1000 Econo-Cap column (dimensions: 25 mm × 0.53 mm, film thickness: 1.2 mM; Alltech, Laarne, Belgium), a flame ionization detector and a split injector.

### 2.4. RNA Isolation and Sequencing (RNA-seq)

We collected 15 mL of *F. prausnitzii* culture at the end of the exponential growth phase (T22), for each condition and replicate, and added it to 30 mL of RNA Protect bacterial reagent (Qiagen, Hilden, Germany) at pH 6. This mixture was immediately vortexed and incubated for 5 min at room temperature. It was then centrifuged at 5000× *g* for 10 min and the pellet was recovered and stored at −80 °C until use. RNA was isolated by enzymatic and mechanical lysis followed by use of the RNAeasy Mini Kit (Qiagen, Hilden, Germany). The RNA was concentrated during DNAse treatment with the RNeasy MiniElute Cleanup kit (Qiagen, Hilden, Germany). Quantity and quality of the RNA was assessed with a NanoDrop^®^ photometer (Thermo Scientific, Waltham, MA, USA) and an Agilent 2100 Bioanalyzer (Agilent Technologies, Palo Alto, CA, USA) respectively. We used 1 µg of DNA-free RNA with a RIN value > 8.0 from each sample for ribosomal RNA removal and library construction with Ribo-zero and the Scriptseq v2 RNAseq Library preparation kit (Illumina, San Diego, CA, USA). Quality control was performed on libraries with the Agilent High-Sensitivity DNA Kit (Agilent Technologies, Palo Alto, CA, USA). The 15 total RNA libraries were sequenced in a single lane on a HiSeq 4000 (Illumina, San Diego, CA, USA), in the 50-cycle single-read configuration, according to the manufacturer’s protocol. We used the HiSeq SBS Kit v3 for sequencing, with HiSeq Control Software 3.3.20 and RTA v2.5.2. Reads in bcl format were demultiplexed with the 6 bp Illumina index and CASAVA 1.8, allowing a single base–pair mismatch per library, and were converted to fastq format with bcl2fastq.

### 2.5. Differential Expression Analysis

The fastQ reads for each condition and replicates were inspected for quality and adapters with CUTADAPT version 1.4.1 [33] and for ribosomal RNA content with SortMeRNA version 2.1 [34]. Clean reads were mapped to the *F. prausnitzii* A2-165 representative genome (GCF_000162015.1) using STAR version 2.6.0a [35]. HTSeq version 0.11.1 was used to quantify the expression of the 2743 Open Reading Frames, and the R Bioconductor tximport package version 1.10.1 to build a count matrix with all the samples. Only transcripts with more than 1 cpm (Counts Per Million) in at least 3 samples were retained. We performed the differential expression analysis with the DESeq2 version 1.22.2 [36] package, with 3 biological replicates for each sample. We applied cutoffs of P-adj (corrected FDR) < 0.05 and absolute log_2_FoldChange (log_2_FC) > 1.5 for the detection of genes differentially expressed between the conditions considered and the negative control.

### 2.6. Functional Annotation

Extended functional annotation was performed, comparing *F. prausnitzii* A2-165 (GCF_000162015.1) ORFs with the sequences in various databases. The December 2014 update of the COG (Cluster of Orthologous Groups of proteins) database [37] was used, together with BLAST(p) version 2.7.1 [38] with a cutoff set at 1 × 10^−6^ for e-value and 40% for identity and coverage, to assign COG ID, functional classes and COG annotations. COG terms were validated through the CD-Search service from NCBI using RPS-Blast with 1 × 10^−6^ as e-value cutoff and the best hits were selected in terms of e-value. Proteins were also scanned for carbohydrate-related domains with the dbCAN2 [39] web server. KEGG Orthology terms and related pathways were identified with the BlastKOALA server [40]. GO terms for the three ontologies were obtained from the *F. prausnitzii* A2-165 page at BioCyc (https://biocyc.org/). As a result of the re-annotation, 1845 ORFs (67.26% of the total number of 2743 ORFs) were annotated with a known function based on the identification of at least one term from any ontology. *F. prausnitzii* A2-165 genome assembly (GCF_000162015.1) was inspected for Lagaffe and Mushu phages [41] with GenBank accession codes MG711461 and MG711460, respectively. Genome assembly proteins were compared to the 65 Lagaffe and 54 Mushu phage proteins using Blastp v2.7.1 with default parameters. Only hits with e-value lower than 1 × 10^−5^, identity higher than 90% and coverage higher than 60% were considered. Hits were then manually curated according to synteny within the phage genome. As a result, 62 and 47 proteins were identified as Lagaffe and Mushu proteins, respectively.

### 2.7. Statistics and Graphs

All statistical analyses were done with R version 3.6. Graphs were plotted with the ggplot2 package (version 3.3.1). Linear regression analyses were performed with the *lm* function of the stats package v 3.6.1 to evaluate the growth curves (OD_600_), the relation butyrate/OD_600_ and the relation butyrate/acetate (note that for *L. lactis* subsp. *cremoris* CNCM I-3558, a sample at T = 22 h was missing). In one of the regression analyses performed (butyrate/acetate), a correction factor based on the time at which determinations were made was introduced into the model. Growth rates were obtained based on linearity during exponential phase. To obtain decay rates, a linear model was fitted following the maximal growth. Differences in growth rate and decay between conditions were tested by Anova followed by Tukey-hsd. Circos version 0.69-6 [42] was used to produce a circular plot that integrates the differential expression analysis and COG annotation data. An overview of the bioinformatic pipeline used in this study is provided in Figure S1.

### 2.8. Access to Data and Codes

Raw RNA-seq fastq files are available at ArrayExpress under project E-MTAB-9387.

## 3. Results and Discussion

### 3.1. Selection of Strains Based on the Growth of F. prausnitzii

Thirteen strains of lactic acid bacteria (*Lactococcus, Streptococcus,* and *Lactobacillus*) and *Bifidobacterium* (Table S1) were tested for their capacity to alter *F. prausnitzii* growth. The effects of the cell-free supernatants from these strains were measured under suboptimal *F. prausnitzii* growth conditions obtained by removal of the vitamin pool from the growth medium. The *F. prausnitzii* growth curves (OD_600_) obtained up to 72 h (Figure S2) allowed us to rank the thirteen strains. We observed that four of them either enhance growth (*L. paracasei* CNCM I-3689) or delay lysis of *F. prausnitzii*: *L. lactis* subsp. *lactis* CNCM I-1631, *L. lactis* subsp. *cremoris* CNCM I-3558, or *S. thermophilus* CNCM I-3862) compared to the control (*F. prausnitzii* alone). We selected these four strains to further investigate the metabolic response of *F. prausnitzii*.

### 3.2. Growth and Metabolic Response of F. prausnitzii to Cell-Free Supernatants

**Figure 1:**
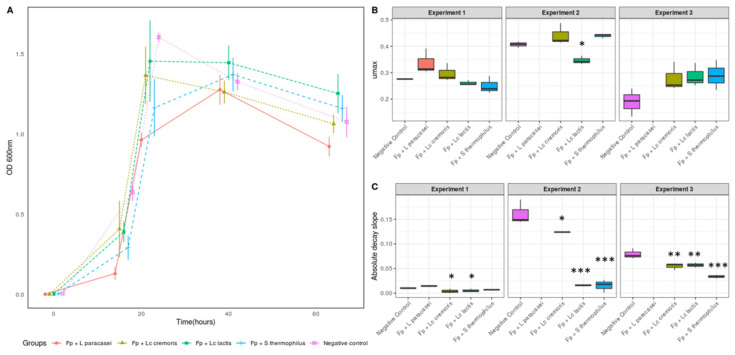
*F. prausnitzii* growth exposed to cell-free supernatants of lactic acid bacteria (**A**) *F. prausnitzii* A2-165 growth, as assessed on the basis of the OD_600_ values for the supernatants with the highest activity (*L. paracasei* CNCM I-3689, *L. lactis* subsp. *cremoris* CNCM I-3558 *(Lc. cremoris)*, *L. lactis* subsp. *lactis* CNCM I-1631 *(Lc. lactis)*, and *S. thermophilus* CNCM I-3862) and the negative control. The mean and SD for replicates are indicated for each timepoint. (**B**) Maximum growth rate (μmax) and (**C**) decay observed of *F. prausnitzii* A2-165 in three independent biological experiments (three technical replicates per experiment). * *p* < 0.05, ** *p* ≤ 0.01, *** *p* ≤ 0.001 versus control group, based on Anova followed by Tukey test.

We co-incubated *F. prausnitzii* with supernatants of the four selected strains for 65 h and sampled at five timepoints (*t* = 0 h (T0), *t* = 16 h (T16), *t* = 22 h (T22), *t* = 40 h (T40), and *t* = 65 h (T65)) (Figure 1A). Maximum growth rate (µmax, ODxh^−1^) was calculated based on the linear phase of exponential growth (Figure 1B)*. F. prausnitzii* exposed to supernatants of *L. paracasei* CNCM I-3689 had a higher mean max growth (0.33) followed by *L*. *lactis* subsp. *cremoris* CNCM I-3558 (0.30), Control (0.27), *L. lactis* subsp. *lactis* CNCM I-1631 (0.26), and *S. thermophilus* CNCM I-3862 (0.25). Decay was measured by linear regression between the time point at which maximum growth was reached and the following time point (Figure 1C). The decay was higher for the control and *L. paracasei* CNCM I-3689, followed by *S. thermophilus* CNCM I-3862, *L. lactis* subsp. *cremoris* CNCM I-3558, and *L. lactis* subsp. *lactis* CNCM I-1631. Experiments were reproduced at least three times and yielded similar conclusions that decay was less pronounced following exposure to bacterial supernatants (especially *S. thermophilus)*, than after exposure to control medium.

Recently, a study reported *L. paracasei* CNCM I-1518 reduced the lysis of *F. prausnitzii* A2-165 compared to the mono-culture [43]. Other studies based on synthetic communities revealed that *F. prausnitzii* grows better in presence of other bacterial strains than alone [44,45]. The identification of metabolites able to sustain the growth of butyrate producers is of particular relevance. A recent in vitro study highlighted the auxotrophy of *F. prausnitzii* for multiple B-vitamins. However, while several auxotrophic butyrate producers for folate or thiamine could benefit from the presence of prototrophic strains, vitamin-independent growth stimulation was observed for *F. prausnitzii* A2-165, suggesting that it used other growth factors from other bacterial members [29].

**Figure 2:**
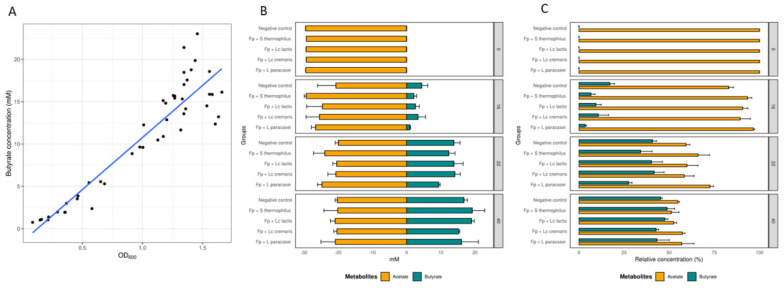
Acetate and butyrate levels of cultures of *F. prausnitzii* exposed to cell-free supernatants. (**A**) Correlation of butyrate production and growth over time (R^2^ = 0.8664, *p*= 2.2 × 10^−16^). (**B**) Acetate consumption and butyrate production (concentrations in mM) and (**C**) proportion at 16, 22, and 40 h of incubation. Measurements were performed for three replicates; the mean and SD are shown.

Butyrate was measured across cell growth. A regression model was used to assess the correlation between butyrate production and growth. A significant correlation was found between butyrate production and bacterial growth, measured in OD units (R = 0.9233, *p*-value = 2.2 × 10^−16^, Figure 2A), consistent with previous findings [14]. Butyrate production in *F. prausnitzii* is driven mainly by consumption of acetate, and to a smaller degree by other carbon sources (such as glucose) [18]. We therefore monitored the kinetics of acetate consumption, and butyrate production over time (Figure 2B). We found a significant correlation between acetate consumption and butyrate production (R = −0.5179768, *p* = 0.0001), consistent with the findings of previous studies [14,46]. After adjustment of the linear regression model for time, we found that about 87% (R^2^ = 0.8664, *p*= 2.2 × 10^−16^) of the butyrate produced was generated through acetate consumption in line with other studies (FEEDAP (2012). The rest of the butyrate production (around 13%) may originate from other sources, such as glucose, as previously reported [18]. At T16, most of the butyrate produced by *F. prausnitzii* originated from acetate for a majority of the supernatants tested. By contrast, *F. prausnitzii* exposed to *S. thermophilus* CNCM I-3862 supernatant produced butyrate mostly via an alternative carbon source pathway (such as glucose) at T16, since no change in acetate concentration was observed (Figure 2B). At the final timepoint (T40), acetate consumption and butyrate production were similar in all conditions (*p* = 0.995 for the acetate and *p* = 0.373 for the butyrate). Similar findings were observed for their relative abundance (Figure 2C). Butyrate level was the highest in *F. prausnitzii* exposed to supernatants of *S. thermophilus* CNCM I-3862 and *L. lactis* subsp. *lactis* CNCM I-1631, with butyrate produced by both acetate consumption and via an alternative pathway.

Previous studies reported enhanced butyrate concentrations by different *F. prausnitzii* strains co-cultured with *Bifidobacterium* sp including *B. adolescentis* L2–32 when grown on starch or fructooligosaccharide [19] and *B. catenulatum*, when grown on fructooligosaccharide [47].

### 3.3. The Transcriptional Response of F. prausnitzii to Cell-Free Supernatants

We then explored the transcriptional response of *F. prausnitzii* exposed to different cell-free supernatants, using RNAseq to identify common and specific pathways modulated by cell-free supernatants. We used samples collected at T22 during the exponential growth phase under all five conditions (four supernatants and control).

We performed quality control of the sequences (removal of rRNA, reads with Q < 20 and short reads) and the high-quality reads (around 22.3 ± 1.97 million clean reads per sample or replicate) were mapped against the representative *F. prausnitzii* A2-165 genome. We were able to map about 98.8 ± 0.46% of the reads onto this genome, of which 85.29 ± 0.98% of reads were mapped against both coding and non-coding annotated RNA elements. However only 63.52 ± 3.57% of the high-quality reads mapped against annotated ORFs in the annotated transcriptome. The fact that almost all the sequences could be mapped onto the genome but that about 15% fewer reads than expected could be mapped onto the whole transcriptome suggests that annotations are still lacking in the GCF_000162015.1 representative genome. Similar observations are found in the literature for other organisms with poorly characterized genomes, such as *Akkermansia muciniphila* [48].

#### 3.3.1. Differential Expression

2607 of the 2743 genes in the genome (95.04%) were included in the differential expression analysis. A principal component analysis (PCA) plot of normalized counts of the genes included for the differential expression analysis showed separation of the transcriptional response of the *F. prausnitzii* control from that of *F. prausnitzii* exposed to supernatants from the four lactic acid bacteria (Figure S3). We chose log2 FC = 1.5, as used in previous studies [49] for detection of differentially expressed genes (DEG). *F. prausnitzii* exposed to cell-free supernatant from *S. thermophilus* CNCM I-3862 displayed the strongest transcriptional response, with 22.4% of its genes differentially expressed, followed by *L. paracasei* CNCM I-3689 (18.8%), *L. lactis* subsp. *lactis* CNCM I-1631 (18.7%), and *L. lactis* subsp *cremoris* CNCM I-3558 (14.1%) (Table S2 and Figure S4). The magnitude of the DEG response was within the range reported by a similar study of another gut bacterium, *Akkermansia muciniphila* [48] (Table S3).

**Figure 3:**
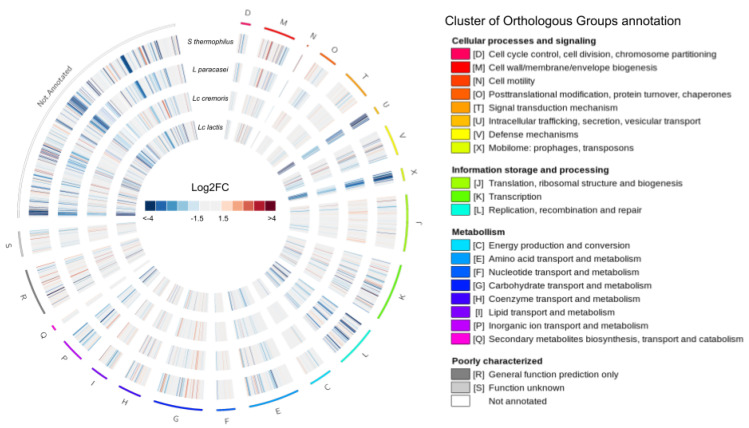
Circular representation of the differentially expressed genes (DEGs) in each of the functional categories. The Cluster of Orthologous Groups of proteins (COG) categories and the log2FC of the genes in each category are indicated. From outside to inside, we have the DEGs for *L. paracasei* CNCM I-3689, *L. lactis* subsp. *lactis* CNCM I-1631, *L. lactis* subsp. *cremoris* CNCM I-3558, and *S. thermophilus* CNCM I-3862.

Figure 3 summarizes the analysis of differential expression. In this circular plot, the DEGs are circumferentially organized according to the COG functional classification.

**Figure 4:**
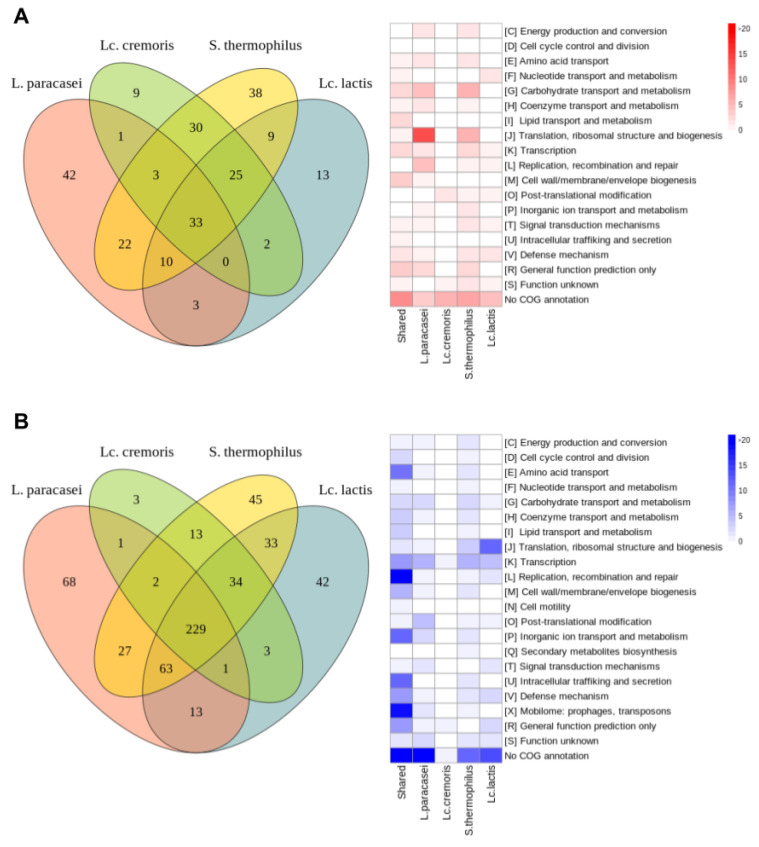
Transcriptional response of *F. prausnitzii* to cell-free supernatants. Venn diagrams of differentially expressed (**A**) upregulated and (**B**) downregulated genes. Gene functions, based on the COG classification, are shown in a heatmap to the right of the Venn diagrams for the upregulated and downregulated genes. The heatmap colors indicate the number of genes for each category of the COG classification.

The four experimental conditions are presented as concentric rings of log2 FC expression levels. COG families that display the largest numbers of DEGs belonged to the J (Translation, ribosomal structure and biogenesis), K (transcription), L (replication, recombination, and repair), G (carbohydrate transport and metabolism), X (mobilome:prophages, transposons), M (cell wall/membrane/envelope biogenesis), U (intracellular trafficking, secretion, and vesicular transport), and E (amino acid transport and metabolism) categories (Figure S5). A more detailed analysis of the DEGs allowed us to identify common genes and functions affected by exposure of *F. prausnitzii* to each of the supernatants as well as specific genes and functions only detected as significant in one of the conditions (Figure 4).

#### 3.3.2. Common Differentially Expressed Genes

We identified 262 genes (9.55% of the 2743 genes in the genome) differentially expressed in all conditions relative to the control (Figure 4). Of these, 33 and 229 were significantly up- and downregulated, respectively. The classification of these genes in COG families revealed that most had no ortholog in the database of annotated genomes, resulting in an unknown/general COG (mostly in the R and S categories) or no annotation (Figure 3). Since some DEG were assigned to known functional categories, we could determine putative impacted functionalities of each of the conditions compared with the control. The most relevant impacted functionalities are described as follows.

- Common upregulated genes

An analysis of the 33 commonly upregulated DEGs revealed several altered associated functions based on COG family annotation when comparing treatments to control: M (cell wall structure and biogenesis, and outer membrane) with four genes, R (general function) four genes, G (carbohydrate metabolism and transport), I (lipid metabolism) and K (transcription) with three genes each, and V (defense mechanisms) with two genes (Table S4), indicating the effect of the supernatants on metabolism, cell division, and defense of *F. prausnitzii*.

Focusing on those genes with COG classifications relating to carbohydrate metabolism, we identified the FAEPRAA2165_RS03315 and FAEPRAA2165_RS02585 genes, both encoding beta-galactosidases involved in the metabolism of lactose into galactose. Previous studies showed that *F. prausnitzii* can metabolize lactose [17]. The MFS transporter annotated FAEPRAA2165_RS03310 gene, and the glycosyltransferase transferase FAEPRAA2165_RS04075, are involved in the putative capsule polysaccharide biosynthesis pathway. Other genes involved in carbohydrate metabolism, including the galactose production pathway and some transporters were affected in all conditions versus control (Table S4).

The significantly up-regulated V genes encoded ABC transporters that might also be involved in other processes, such as capsule polysaccharide biosynthesis, in which they facilitate the transport of molecules. We identified others involved in the putative capsule polysaccharide biosynthesis pathway (FAEPRAA2165_RS04065, a glycosyltransferase) or promoting changes in cell wall composition (FAEPRAA2165_RS09205, D-alanyl-D-alanine carboxypeptidase and FAEPRAA2165_RS09720, N-acetylmuramoyl-L-alanine amidase), suggesting possible modification of the *F. prausnitzii* cell wall. The cell wall can be remodeled by bacterial hydrolases, amidase, glycosidase (N-acetylglucosaminidases, and lysozymes, or N-acetylmuramidases), or peptidase whose activities are carefully regulated to maintain cell integrity or lead to bacterial lysis [50].

Seven other genes related to carbohydrate metabolism are also involved in the capsule polysaccharide biosynthesis pathway (cell wall, as suggested by Heinken and coworkers [17] (FAEPRAA2165_RS14910, FAEPRAA2165_RS04025, FAEPRAA2165_RS04045, FAEPRAA2165_RS04065, FAEPRAA2165_RS04075, FAEPRAA2165_RS10175, and FAEPRAA2165_RS10180). However, we did not observe a capsule-like structure using India ink staining (data not shown) suggesting that this pathway might be related to other cell wall-related structures. All seven annotated elements had a FC ≥ 1 in all conditions compared to the control, but only some of them were significantly up-regulated (with >1.5 FC and significant p-value, especially in *L. paracasei* CNCM I-3689 and *S. thermophilus* CNCM I-3862 supernatants).

The up-regulation of the genes in the putative capsule polysaccharide biosynthesis pathway indicates the relevant role of the cell wall in the stability of the bacteria. The large number of elements related to sugar transport and the aforementioned capsule polysaccharide biosynthesis pathway found to be mainly upregulated, may suggest an increase in the stability of the cell in response to stress, as described in other bacteria [51,52]. Little is currently known about the structure and composition of the cell wall of *F. prausnitzii*, suggested to either lack lipopolysaccharides (LPS) or have an unusual LPS composition [53,54]. Our results indicate that the cell wall of *F. prausnitzii* merits further study, especially as the extracellular matrix has been reported to play a role in abolishing the inflammatory response and inflammation on exposure to cultures of dendritic cells and in the mouse model of IBD, respectively [10].

- Common downregulated genes

About 49.3% of the 229 genes that were downregulated have no known function or are poorly characterized (113 of the 229 genes) (Table S5). The remaining 50.7% of the genes commonly downregulated were related to replication, recombination and repair (COG family L: 23 genes), mobilome (COG family X: 19 genes), inorganic ion transport, and metabolism (COG family P: 12 genes), intracellular trafficking and secretion (COG family U: 12 genes), or amino-acid transport and metabolism (COG family E: 11 genes). Those ORFs involved in the cell division machinery appeared to be less strongly down-regulated than in control conditions compared with other COG category, indicating that the cell adjusted its metabolism towards other functions, such as cell wall biogenesis. Several genes were annotated as TraG and TraD conjugal protein transfer ATPase genes and they were distributed along the *F. prausnitzii* genome, as previously reported [16]. All of them were found to be downregulated, indicating that secretion and vesicular transport (COG family U) were affected, possibly because these functions are not required at this stage and metabolic efforts are focused on other functions such as extracellular matrix formation. Genes participating in the phage cycle, such as those encoding portal proteins, tail proteins, or integrases are annotated as belonging to the ‘[X] mobilome: phages and transposons’ COG category. They appeared to be commonly downregulated in all four sets of conditions (Table S5). *F. prausnitzii* is thought to make frequent use of the proteins encoded by these genes, as large numbers of such elements are present in its pangenome [16]. Even though some of these elements are incomplete and inactive, they may play a role in bacterial dynamics through plasticity, by integrating genes from other genomes. Two complete prophages have been described for *F. prausnitzii* A2-165 (Lagaffe and Mushu). Comparative analysis of the *F. prausnitzii* A2-165 assembly (GCF_000162015.1) and the reference genomes for both phages revealed that genes ranging from FAEPRAA2165_RS07765 to FAEPRAA2165_RS08075 were assigned to the Lagaffe phage while those from FAEPRAA2165_RS14080 to FAEPRAA2165_RS14325 to the Mushu phage. Interestingly, most of the Lagaffe annotated elements followed the same pattern of down-regulation across all the tested probiotics when compared to the control (39 genes detected as downregulated). We did not find any DEGs commonly downregulated related to the Mushu phage, we only found the transposase FAEPRAA2165_RS14120 down-regulated for the *S. thermophilus* CNCM I-3862 condition. Interestingly, the observation of reduced mobilome may be relevant as there is an increasing body of evidence that phages are involved in the dynamics of gut microbiota [55] and recent studies call attention to the large number of phages of *F. prausnitzii* [41].

Overall, these results suggest that the genes commonly upregulated are mostly involved in cell-wall production and modification, whereas those involved in the cell cycle and replication and in the mobilome (phages and transposons) are commonly downregulated, with a specific role for the Lagaffe phage that seems to be less active in all conditions compared with the control culture, consistent with the lower lysis observed.

#### 3.3.3. Specific Differentially Expressed Genes

*F. prausnitzii* also displayed a specific transcriptome response to bacterial supernatant exposure; specific up- and down-regulated genes designate those elements that are found only significantly up- or down-regulated in one of the four conditions compared to the control condition (Table S6). Despite *S. thermophilus* supernatant induced the largest number of total DEGs, *L. paracasei* CNCM I-3689 supernatant induced the strongest specific significant response among all the tested supernatants. In short, *F. prausnitzii* answer to the exposure of *L. paracasei* CNCM I-3689 supernatant, may stabilize cell wall formation, through the upregulation some genes involved mainly in peptidoglycan formation, and the inhibition of cell wall degradation genes, as well as reduced stress response due to the repression of several stress-related transcriptional regulators. *F. prausnitzii* exposed to supernatant of *S. thermophilus* CNCM I-3862 related to mobilome, defense mechanism based on transporters and cell replication/transcription functions, were more strongly downregulated in this condition than in the others tested, suggesting that the repression of the mobilome could be more pronounced in *F. prausnitzii* exposed to *S. thermophilus* CNCM I-3862 supernatant, in line with the lower decay rate observed for the growth curve. While reduced decay observed following *L. paracasei* CNCM I-3689 might be associated with higher expression of genes related to extracellular matrix, higher repression on mobilome and proteins related to stress response such as chaperones was observed for *S. thermophilus* CNCM I-3862. *F. prausnitzii* response to *L. lactis* subsp. *lactis* CNCM I-1631 suggests a higher stress response with repressed autophagy genes and chaperone buffering protein. Less specific response was observed for *L. lactis* subsp. *cremoris* CNCM I-3558.

## 4. Conclusions

We have explored the metabolic response of *F. prausnitzii* to supernatants of a few lactic acid bacteria. Common responses included decrease in expression of genes related to the activity of mobile elements or involved in cell division and increase in expression of genes related to the formation of an extracellular polymer matrix. Our results collectively suggest that metabolites resulting from lactic acid bacteria may modify the metabolism of *F. prausnitzii*. The responses observed by RNA-seq are consistent with the reduction of decay observed in *F. prausnitzii* exposed to lactic acid bacteria supernatants. Given the increasing evidence that *F. prausnitzii* is beneficial to human health, the identification of factors altering its maintenance, both in vitro and *in vivo*, is of great interest. Finally, our study highlights the relevance of the secretome of lactic acid bacteria as nutritional source for gut microbes. Our findings constitute a first step toward approaches for modulating the growth and metabolic response of *F. prausnitzii* and pave the way for further high-throughput approaches combined with continuous bacterial growth kinetics.

## 5. Patents

MD, PG, and ML have a patent application related to this work (International Application No.PCT/EP2015/081148).

## Supplementary Materials

The following are available online at https://www.mdpi.com/2076-2607/8/10/1528/s1, **Figure S1:** Overview of the bioinformatics pipeline used in this study, **Figure S2:** *F. prausnitzii* A2-165 growth, as assessed on the basis of the OD_600_ values for the supernatants with 13 lactic acid bacteria/*Bifidobacterium* strains. **Figure S3:** Principal component analysis (PCA) plot of normalized counts of the genes included for the analysis of differential expression for *F. prausnitzii* exposed to control or cell-free supernatants from the four lactic acid bacteria. **Figure S4:** Volcano plots representing the differential expression analysis of *F. prausnitzii* A2-165 genes following exposure to cell-free supernatants. Genes are considered to be differentially expressed in DESeq2 analysis when absolute log2 FoldChange (log 2FC) > 1.5 (red dots). Genes with a significant p-value but a log2 FC < 1.5 are indicated by blue dots and were not considered in the analysis, and genes for which the p-value was not significant are indicated by black dots. Dashed lines indicate the threshold adjusted p-value (y axis) and log2FC (x-axis) for a gene to be classified as indicated above. **Figure S5:** Bar chart of the number of genes found to be differentially expressed in each condition, classified by COG family. The numbers of genes identified as up- and downregulated are shown in blue and red, respectively. **Table S1.** List of strains used to generate supernatants and of the commensal strain on which the supernatant was tested. **Table S2.** Genes identified as differentially expressed (with a log2 FC > 1.5) and the percentage of the total of 2607 genes included in the analysis displaying differential expression in each condition relative to the negative control. The numbers of genes upregulated and downregulated, the total number of genes and the percentage are indicated. **Table S3.** Genes identified as differentially expressed (with a log2 FC > 2) and the percentage of the total of 2607 genes included in the analysis displaying differential expression in each condition relative to the negative control. The numbers of genes upregulated and downregulated, the total number of genes and the percentage are indicated. **Table S4.** List of commonly upregulated genes and COG functions. The gene ID, the log2 FC for each of the conditions relative to the negative control, the closest annotated product in the COG database and the COG category of the gene based on the closest annotated product in the COG database are indicated. **Table S5.** List of commonly downregulated genes and COG functions. The gene ID, the log2 FC for each of the conditions relative to the negative control, the closest annotated product in the COG database and the COG category of the gene based on the closest annotated product in the COG database are indicated. **Table S6.** List of specific differentially expressed genes (up- and downregulated) and COG functions for each condition. Each sheet shows the gene ID, the log2 FC relative to the negative control, the closest annotated product in the COG database and the COG category of the gene based on the closest annotated product in the COG database and the COG term for each of the conditions.
