# Supplementary figures and images for: Metabolic Response of Faecalibacterium prausnitzii to Cell-Free Supernatants from Lactic Acid Bacteria
Source: Microorganisms. 2020 Oct 5;8(10):1528. doi: 10.3390/microorganisms8101528 (PMC7650636; doi:10.3390/microorganisms8101528)

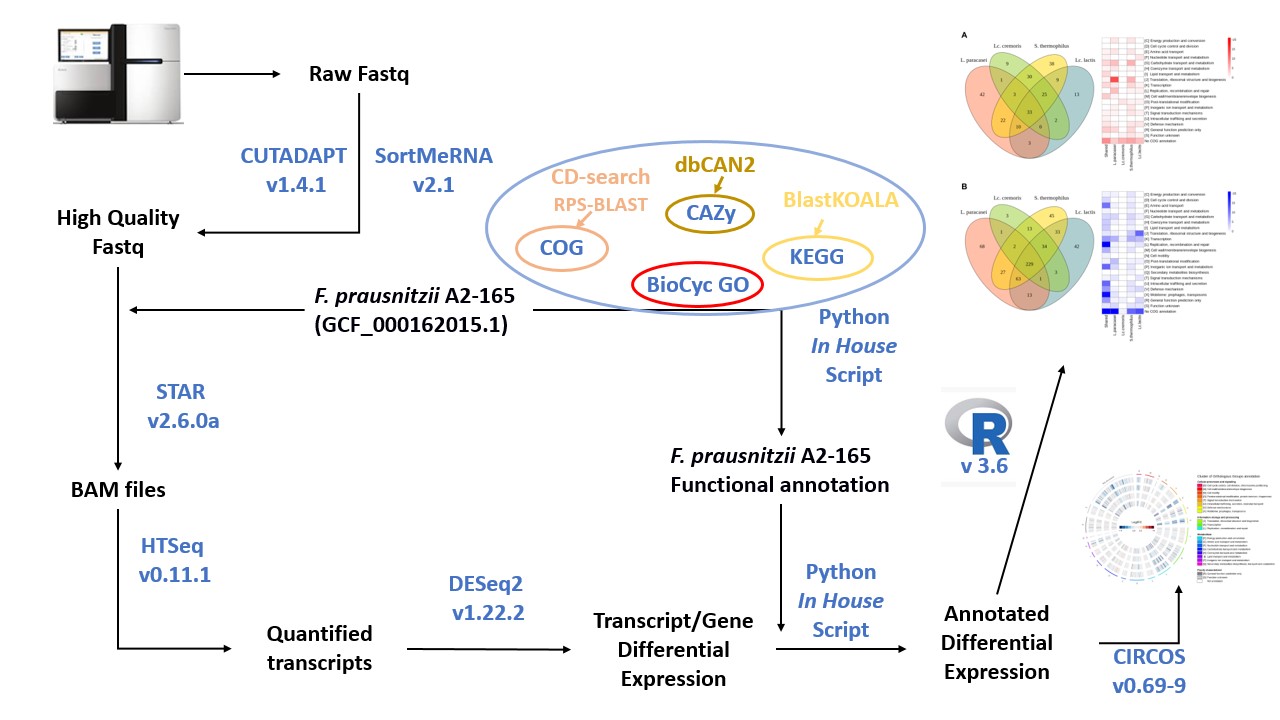

Supplement: Supplementary file 1 [file microorganisms-08-01528-s001.zip › Figure_S1.jpg]

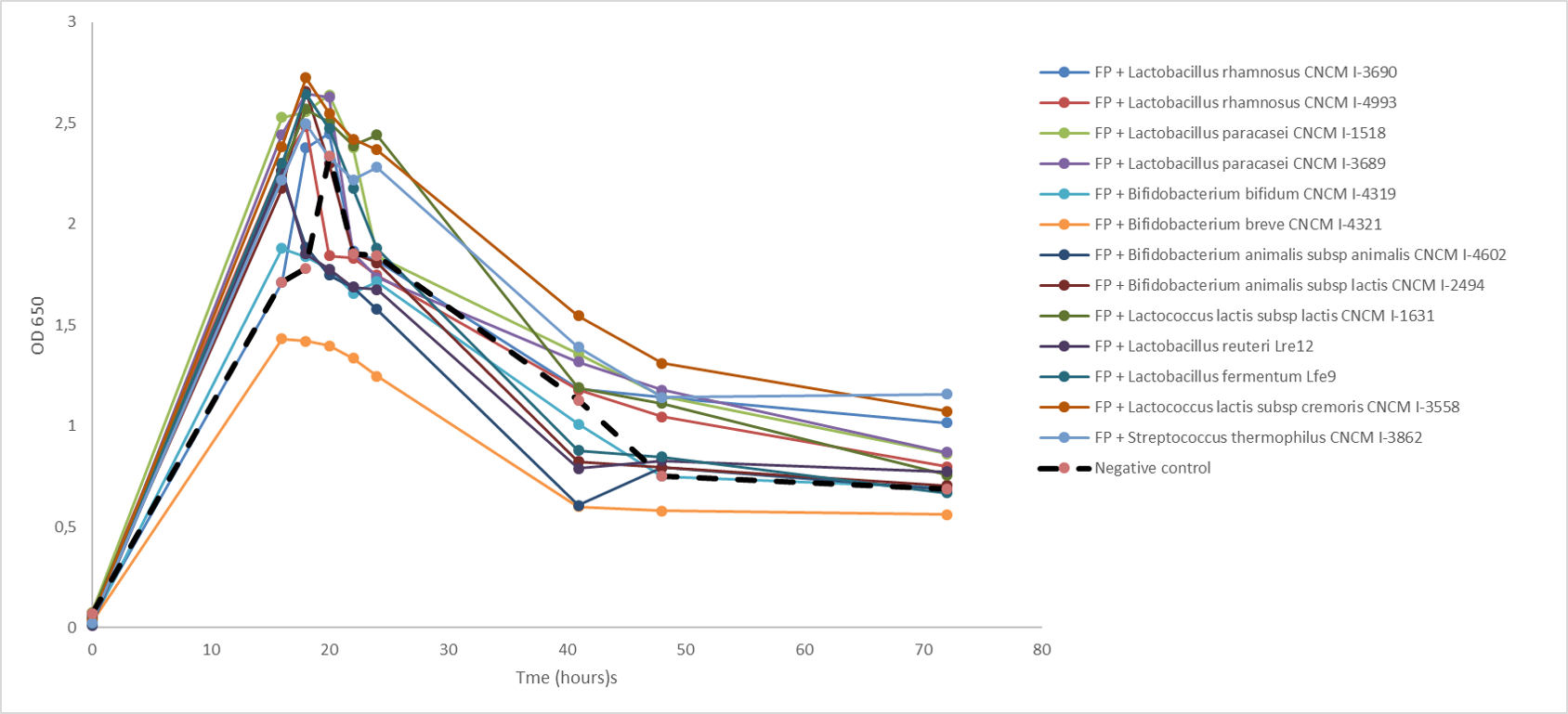

Supplement: Supplementary file 1 [file microorganisms-08-01528-s001.zip › Figure_S2.png]

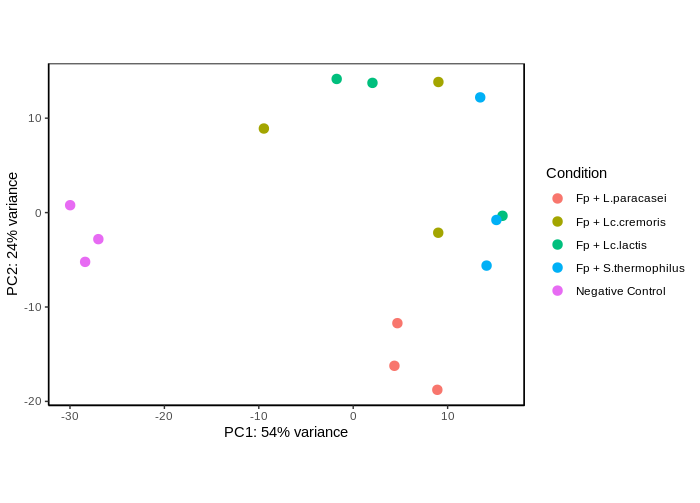

Supplement: Supplementary file 1 [file microorganisms-08-01528-s001.zip › Figure_S3.png]

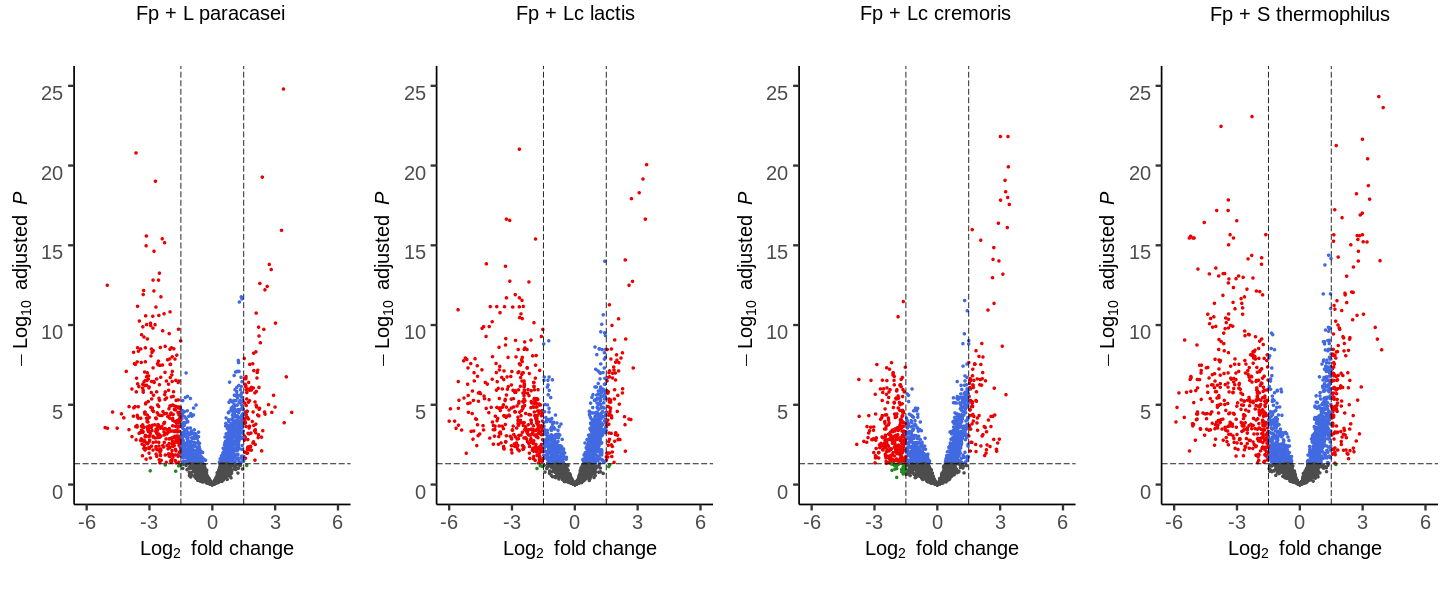

Supplement: Supplementary file 1 [file microorganisms-08-01528-s001.zip › Figure_S4.png]

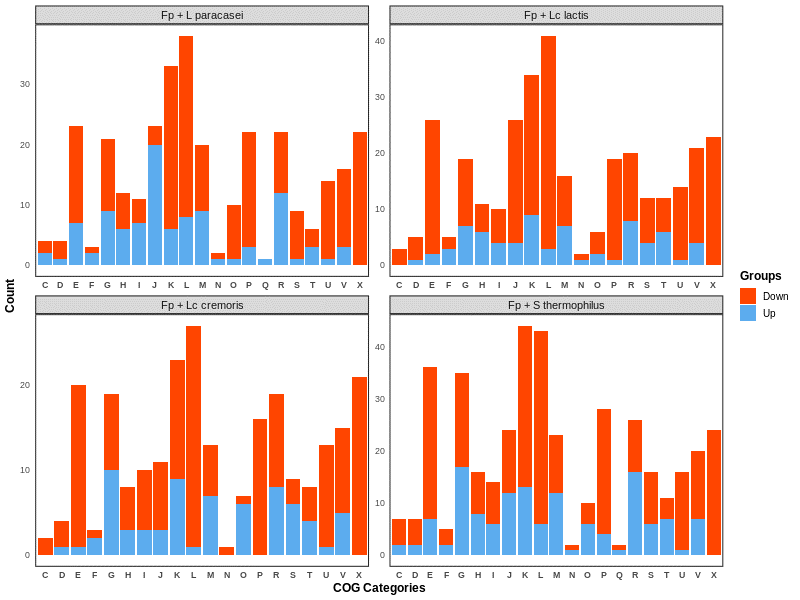

Supplement: Supplementary file 1 [file microorganisms-08-01528-s001.zip › Figure_S5.png]
